# Supplementary material for: mRNA codon optimization with quantum computers
Source: PLoS One. 2021 Oct 29;16(10):e0259101. doi: 10.1371/journal.pone.0259101 (PMC8555812; doi:10.1371/journal.pone.0259101)
Supplement: S1 File — (DOCX) [file pone.0259101.s001.docx]

# mRNA codon optimization with quantum computers

Dillion M. Fox^1^, Kim M. Branson^2^, Ross C. Walker^1,3^

^1^Data and Computational Science, Medicinal Sciences and Technology, GlaxoSmithKline, 1250 S. Collegeville Rd, Collegeville, PA, 19426, USA

^2^Artificial Intelligence and Machine Learning, Medicinal Sciences and Technology, GlaxoSmithKline, 1250 S. Collegeville Rd, Collegeville, PA, 19426, USA

^3^Department of Chemistry and Biochemistry, University of California San Diego, La Jolla, CA, 92130, USA

# Supplementary Information

### GC-content derivation

We use the following objective function to measure optimality of GC-content:

$$\Delta=\left( \rho_{GC}-\rho_{T} \right)^{2} \left( S1 \right),$$

where $\rho_{T}\epsilon\left[ 0,1 \right]\mathbb{\subset R}$ is the target GC-content,

$$\rho_{GC}=\frac{1}{N}\sum_{i}^{N} s_{i}q_{i} \left( S2 \right)$$

is the GC-content of the nucleotide sequence, $q_{i}\in\{0,1\}$ denotes the value of qubit *i*, and *s_i_* is the calculation of the normalized GC-content for each codon. Equation (S1) is rewritten as a two-body Hamiltonian, $\mathcal{H}_{GC}$, by expanding the terms and substituting the definition of *ρ_GC_* from equation (S2):

$$\mathcal{H}_{GC}\propto\left( \rho_{GC}-\rho_{T} \right)^{2}=\rho_{GC}^{2}-2\rho_{GC}\rho_{T}+\rho_{T}^{2}$$

$$=\left( \frac{1}{N}\sum_{i}^{N} s_{i}q_{i} \right)^{2}-2\rho_{T}\frac{1}{N}\sum_{i}^{N} s_{i}q_{i}+\rho_{T}^{2}$$

$$=\frac{1}{N^{2}}\left( \sum_{i}^{N} s_{i}q_{i} \right)\left( \sum_{j}^{N} s_{j}q_{j} \right)-\frac{2\rho_{T}}{N}\sum_{i}^{N} s_{i}q_{i}+\rho_{T}^{2} \left( S3 \right).$$

The two terms in parentheses in equation (S3) can be rewritten as an outer product. To see this, explicitly write out the terms in the sums and multiply the terms in parentheses:

$$=\frac{1}{N^{2}}\left( s_{0}q_{0}+s_{1}q_{1}+\ldots\right)\left( s_{0}q_{0}+s_{1}q_{1}+\ldots\right)-\frac{2\rho_{T}}{N}\sum_{i}^{N} s_{i}q_{i}+\rho_{T}^{2}$$

$$=\frac{1}{N^{2}}\left( s_{0}q_{0}s_{0}q_{0}+s_{1}q_{1}s_{0}q_{0}+s_{1}q_{1}s_{0}q_{0}\ldots\right)-\frac{2\rho_{T}}{N}\sum_{i}^{N} s_{i}q_{i}+\rho_{T}^{2}$$

The terms in parentheses include all combinations of indices, which is compactly written as an outer product:

$$=\frac{1}{N^{2}}\sum_{i}^{N} \sum_{j}^{N} \left( s\bigotimes s \right)_{ij}q_{i}q_{j}-\frac{2\rho_{T}}{N}\sum_{i}^{N} s_{i}q_{i}+\rho_{T}^{2} \left( S4 \right)$$

Note, this expression strongly resembles the gyration tensor which could be leveraged to compute properties describing the distribution of G’s and C’s in the sequence. The double sum needs to be restricted to only include terms from the upper triangular elements of the matrix. This is achieved by recognizing the symmetry in matrix; the lower triangular and upper triangular elements are equal, so the sum over the upper triangular terms multiplied by two accounts for all off-diagonal terms.

$$\sum_{i}^{N} \sum_{j\neq i}^{N} \left( s\bigotimes s \right)_{ij}q_{i}q_{j})=2\sum_{i}^{N} \sum_{j<i}^{N} s_{i}s_{j}q_{i}q_{j}+\sum_{i}^{N} s_{i}^{2}q_{i}$$

The sum over the diagonal elements is called the trace, and can be written as a single sum:

$$Tr\left[ \sum_{i}^{N} \sum_{j}^{N} \left( s\bigotimes s \right)_{ij}q_{i}q_{j}) \right]=\sum_{i}^{N} s_{i}^{2}q_{i}$$

Note, because *q_i_* is restricted to 0 or 1, it is idempotent with itself, so q_i_*q_i_ = q_i_. A tunable constant is introduced to set the relative importance of this term to compared others. Thus equation (15) from the main text is recovered:

$$\mathcal{H}_{GC}=2c_{GC}\sum_{i}^{N} \sum_{j<i}^{N} \left( s\bigotimes s \right)_{ij}q_{i}q_{j}+c_{GC}\sum_{i}^{N} s_{i}^{2}q_{i}-2\rho_{T}c_{GC}\sum_{i}^{N} s_{i}q_{i}+{c_{GC}\rho}_{T}^{2} \left( S5 \right)$$

### BQM challenges and limitations

Mapping codon optimization to a BQM introduces invalid states to the solution space. A set of constraints are introduced to limit the probability of accessing these invalid states, but quantum devices are subject to noise and therefore invalid states cannot be avoided altogether. Let *S_c_* represent the size of the valid solution space, and let

$$\vec{n}=\left( n_{0},\ldots,n_{N} \right) \left( S6 \right)$$

represent the number of possible codons for each position in a polypeptide sequence of length *N*. The size of the solution space is given by the product of the elements of $\vec{n}$:

$$S_{c}=\prod_{i}^{N} n_{i} \left( S7 \right).$$

The formulation of the BQM maps every codon that can map to the polypeptide sequence to a qubit, which can be read as either a “0” or a “1”. The total size of the BQM solution space, *S_q_*, is then given by:

$$S_{q}=2^{\sum_{i}^{N} n_{i}} \left( S8 \right).$$

Rewriting equation (S8) as a product yields:

$$S_{q}=\prod_{i}^{N} 2^{n_{i}} \left( S9 \right).$$

Thus, the ratio of valid states to invalid states in the BQM, *f_v_*, decreases exponentially as a function of *N*:

$$f_{v}=\frac{S_{c}}{S_{q}}=\prod_{i}^{N} n_{i}2^{-n_{i}} \left( S10 \right).$$

To illustrate the consequences of this expanded solution space, consider a sequence containing 10 methionine residues, which each map to one codon. Then the total solution space only contains 1 valid solution:

$$S_{c}\left( MMMMMMMMMM \right)=\prod_{i}^{N} 1=1.$$

However, the BQM solution space contains many results:

$$S_{q}\left( MMMMMMMMMM \right)=\prod_{i}^{N} 2^{1}=2^{10}.$$

In this case, the BQM is faced with finding the one valid solution in a space containing 2^10^ possibilities. Now consider a special case in which each residue in a sequence of length *N* maps to 4 codons, which is approximately equal to the weighted average of amino acid to codon mappings. In this case,

$$f_{v}\left( 4 codons per position \right)=\frac{S_{c}}{S_{q}}=\frac{\prod_{i}^{N} 4}{\prod_{i}^{N} 2^{4}}=\frac{2^{2N}}{2^{4N}}=\frac{1}{2^{2N}}=\frac{1}{S_{c}}.$$

For each valid state in the BQM, there are 2^2N^ invalid states, which is equal in size to the valid solution space itself. In practice, this means perturbations due to noise are highly likely to push the system into an invalid state which can be difficult to recover from since there are exponentially more invalid states compared to valid states.

### Genetic Algorithm Validation

The GA was run on 72 peptide fragments derived from A0A2U1LIM9 of length 10 and the lowest value was compared to the true ground state by exhaustively scoring all possible nucleotide sequences in the solution space. The number of iterations required to identify the global minimum was recorded for each sequence, and the data was plotted as a histogram in Supplementary Figure S3. The maximum number of required steps to reach convergence was 19. Small peptide fragments were therefore each run 20 times for 100 iterations to ensure that the global minimum was identified for each case.

The number of required iterations increases as a function of sequence length. To heuristically determine the number of iterations required to asymptotically converge full-length sequences (100-1,000 amino acids), protein A0A2U1LIM9 (the longest sequence in the test set) was simulated 10 times with 15,000 iterations each (Supplementary Figure S4). The calculation was determined to asymptotically converge within 6,000 iterations.

## Supplementary Figures


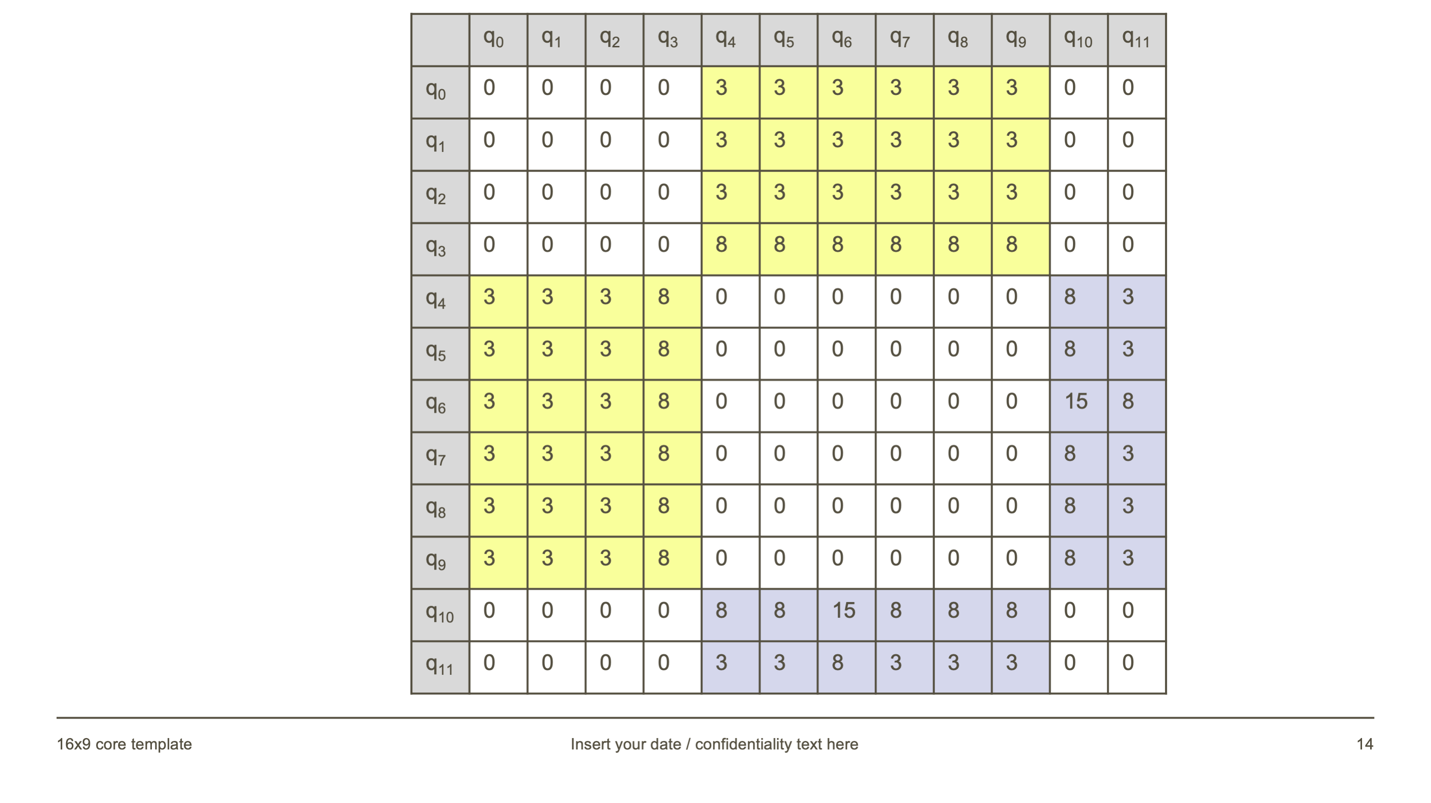


Supplementary Figure S1. Result of applying equation (9) from the main text to the example system shown in Figure 1 from the main text. Positions highlighted in yellow represent couplings between the first and second codon, and lavender represents couplings between the second and third codons.


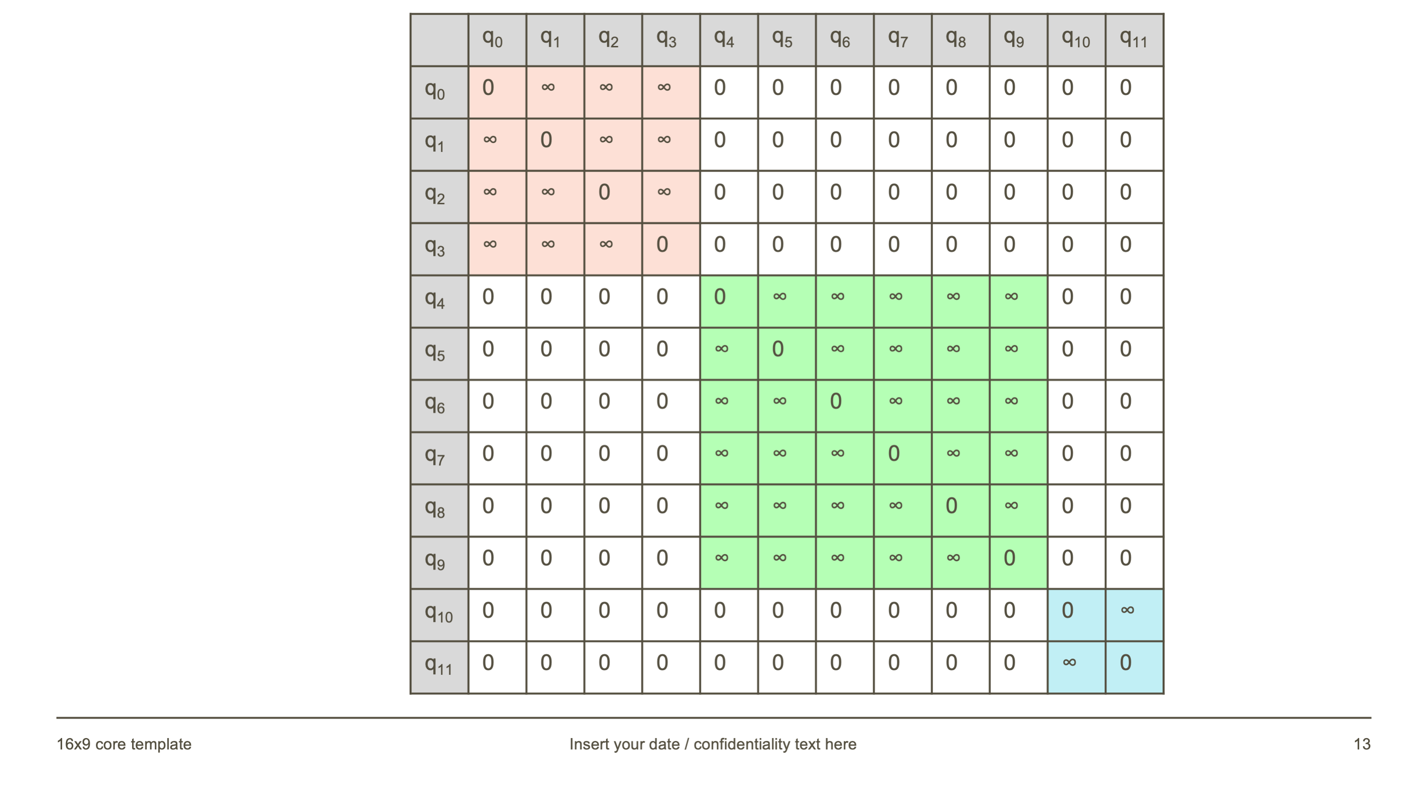


Supplementary Figure S2. Application of **𝛿**’ function (equation (12) from main text) to the example system shown in Figure 1 from the main text. The codons mapping to the same sequence position are highlighted in orange, green, and blue for positions 1, 2, and 3, respectively.


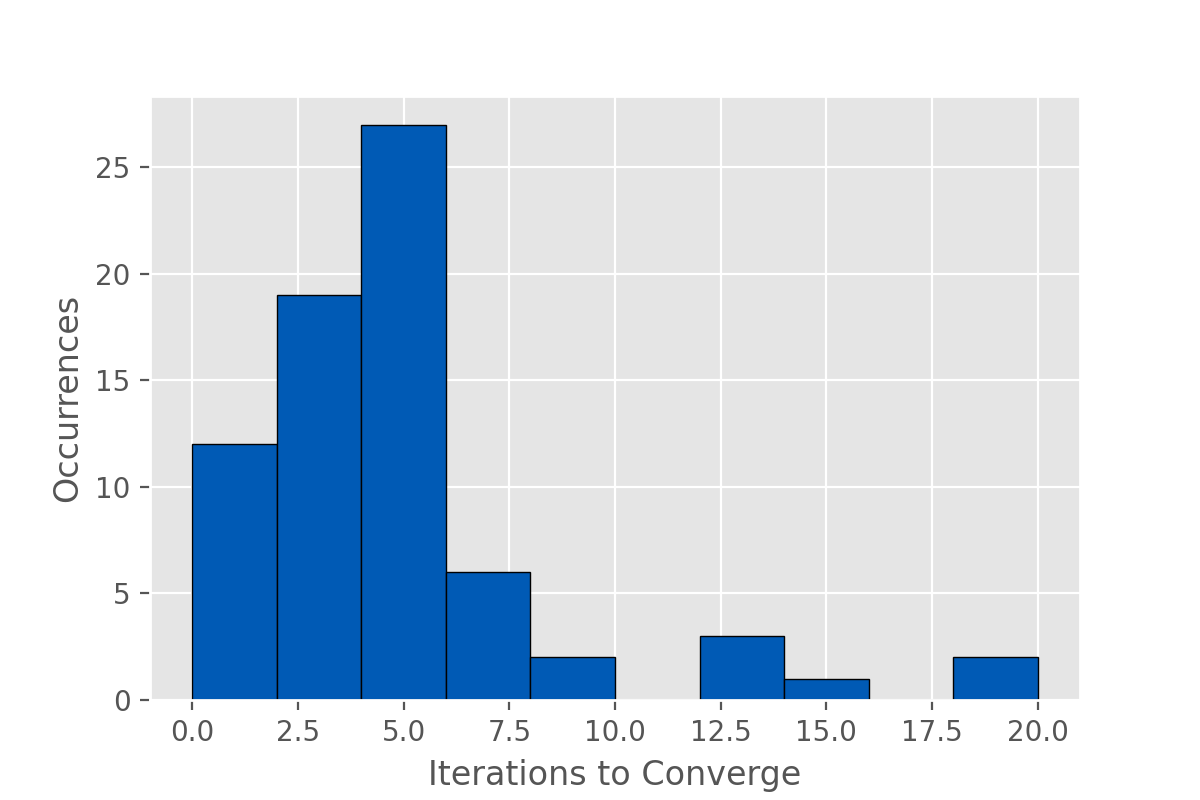


Supplementary Figure S3. Required number of iterations for 72 peptide fragments of length 10 from A0A2U1LIM9 to converge. Convergence was verified by exhaustively enumerating all possible nucleotide sequences and saving the lowest score.


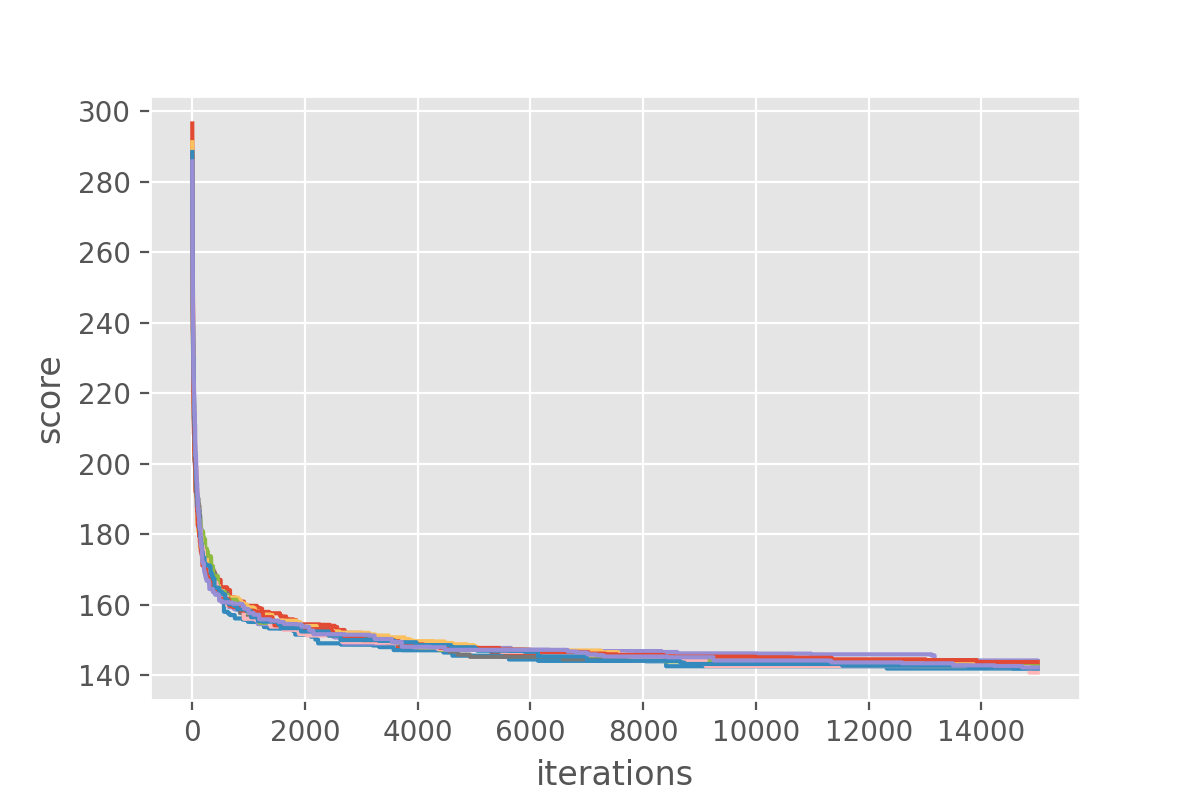


Supplementary Figure S4. Score vs number of GA iterations for protein sequence A0A2U1LIM9, which has 704 amino acids.

## Protein Sequences

>sp|P0DTC2|SPIKE_SARS2 Spike glycoprotein OS=Severe acute respiratory syndrome coronavirus 2 OX=2697049 GN=S PE=1 SV=1

MFVFLVLLPLVSSQCVNLTTRTQLPPAYTNSFTRGVYYPDKVFRSSVLHSTQDLFLPFFS

NVTWFHAIHVSGTNGTKRFDNPVLPFNDGVYFASTEKSNIIRGWIFGTTLDSKTQSLLIV

NNATNVVIKVCEFQFCNDPFLGVYYHKNNKSWMESEFRVYSSANNCTFEYVSQPFLMDLE

GKQGNFKNLREFVFKNIDGYFKIYSKHTPINLVRDLPQGFSALEPLVDLPIGINITRFQT

LLALHRSYLTPGDSSSGWTAGAAAYYVGYLQPRTFLLKYNENGTITDAVDCALDPLSETK

CTLKSFTVEKGIYQTSNFRVQPTESIVRFPNITNLCPFGEVFNATRFASVYAWNRKRISN

CVADYSVLYNSASFSTFKCYGVSPTKLNDLCFTNVYADSFVIRGDEVRQIAPGQTGKIAD

YNYKLPDDFTGCVIAWNSNNLDSKVGGNYNYLYRLFRKSNLKPFERDISTEIYQAGSTPC

NGVEGFNCYFPLQSYGFQPTNGVGYQPYRVVVLSFELLHAPATVCGPKKSTNLVKNKCVN

FNFNGLTGTGVLTESNKKFLPFQQFGRDIADTTDAVRDPQTLEILDITPCSFGGVSVITP

GTNTSNQVAVLYQDVNCTEVPVAIHADQLTPTWRVYSTGSNVFQTRAGCLIGAEHVNNSY

ECDIPIGAGICASYQTQTNSPRRARSVASQSIIAYTMSLGAENSVAYSNNSIAIPTNFTI

SVTTEILPVSMTKTSVDCTMYICGDSTECSNLLLQYGSFCTQLNRALTGIAVEQDKNTQE

VFAQVKQIYKTPPIKDFGGFNFSQILPDPSKPSKRSFIEDLLFNKVTLADAGFIKQYGDC

LGDIAARDLICAQKFNGLTVLPPLLTDEMIAQYTSALLAGTITSGWTFGAGAALQIPFAM

QMAYRFNGIGVTQNVLYENQKLIANQFNSAIGKIQDSLSSTASALGKLQDVVNQNAQALN

TLVKQLSSNFGAISSVLNDILSRLDKVEAEVQIDRLITGRLQSLQTYVTQQLIRAAEIRA

SANLAATKMSECVLGQSKRVDFCGKGYHLMSFPQSAPHGVVFLHVTYVPAQEKNFTTAPA

ICHDGKAHFPREGVFVSNGTHWFVTQRNFYEPQIITTDNTFVSGNCDVVIGIVNNTVYDP

LQPELDSFKEELDKYFKNHTSPDVDLGDISGINASVVNIQKEIDRLNEVAKNLNESLIDL

QELGKYEQYIKWPWYIWLGFIAGLIAIVMVTIMLCCMTSCCSCLKGCCSCGSCCKFDEDD

SEPVLKGVKLHYT

>sp|P07711|CATL1_HUMAN Procathepsin L OS=Homo sapiens OX=9606 GN=CTSL PE=1 SV=2

MNPTLILAAFCLGIASATLTFDHSLEAQWTKWKAMHNRLYGMNEEGWRRAVWEKNMKMIE

LHNQEYREGKHSFTMAMNAFGDMTSEEFRQVMNGFQNRKPRKGKVFQEPLFYEAPRSVDW

REKGYVTPVKNQGQCGSCWAFSATGALEGQMFRKTGRLISLSEQNLVDCSGPQGNEGCNG

GLMDYAFQYVQDNGGLDSEESYPYEATEESCKYNPKYSVANDTGFVDIPKQEKALMKAVA

TVGPISVAIDAGHESFLFYKEGIYFEPDCSSEDMDHGVLVVGYGFESTESDNNKYWLVKN

SWGEEWGMGGYVKMAKDRRNHCGIASAASYPTV

>sp|P49350|FPPS_ARTAN Farnesyl pyrophosphate synthase OS=Artemisia annua OX=35608 GN=FPS1 PE=1 SV=2

MSSIDLKSKFLKVYDTLKSELINDPAFEFDDDSRQWIEKMLDYNVPGGKLNRGLSVVDSY

QLLKGGELSDDEIFLSSALGWCIEWLQAYFLVLDDIMDESHTRRGQPCWFRLPKVGMIAA

NDGILLRNHVPRILKKHFRGKPYYVDLVDLFNEVEFQTASGQMIDLITTLVGEKDLSKYS

LSIHRRIVQYKTAYYSFYLPVACALLMFGEDLDKHVEVKNVLVEMGTYFQVQDDYLDCFG

APEVIGKIGTDIEDFKCSWLVVKALELANEEQKKVLHENYGKKDPASVAKVKEVYHTLNL

QAVFEDYEATSYKKLITSIENHPSKAVQAVLKSFLGKIYKRQK

>sp|A0A2U1Q018|ADH1_ARTAN Alcohol dehydrogenase 1 OS=Artemisia annua OX=35608 GN=ADH1 PE=1 SV=1

MAQKAPGVITCKAAVVWELGGPVVLEEIRVDPPKASEVRIKMLCASLCHTDVLCTKGFPI

PLFPRIPGHEGVGVIESVGKDAKGLKPGDIVMPLYLGECGQCLNCKTGKTNLCHVYPPSF

SGLMNDGTSRMSIARTGESIYHFASCSTWTEYAVADCNYVLKINPKISYPHASFLSCGFT

TGFGATWRETQVSKGSSVAVFGIGTVGLGVIKGAQLQGASKIIGVDVNQYKAAKGKVFGM

TDFINPKDHPDKSVSELVKELTHGLGVDHCFECTGVPSLLNEALEASKIGIGTVVPIGAG

GEASVAINSLILFSGRTLKFTAFGGVRTQSDLPVIIDKCLNKEIQLDELLTHEIHLDNIQ

EAFEILKKPDCVKILIKF

>sp|P35613-2|BASI_HUMAN Isoform 2 of Basigin OS=Homo sapiens OX=9606 GN=BSG

MAAALFVLLGFALLGTHGASGAAGTVFTTVEDLGSKILLTCSLNDSATEVTGHRWLKGGV

VLKEDALPGQKTEFKVDSDDQWGEYSCVFLPEPMGTANIQLHGPPRVKAVKSSEHINEGE

TAMLVCKSESVPPVTDWAWYKITDSEDKALMNGSESRFFVSSSQGRSELHIENLNMEADP

GQYRCNGTSSKGSDQAIITLRVRSHLAALWPFLGIVAEVLVLVTIIFIYEKRRKPEDVLD

DDDAGSAPLKSSGQHQNDKGKNVRQRNSS

>sp|Q1PS23|AMO_ARTAN Amorpha-4,11-diene 12-monooxygenase OS=Artemisia annua OX=35608 GN=CYP71AV1

PE=1 SV=2

MKSILKAMALSLTTSIALATILLFVYKFATRSKSTKKSLPEPWRLPIIGHMHHLIGTTPH

RGVRDLARKYGSLMHLQLGEVPTIVVSSPKWAKEILTTYDISFANRPETLTGEIVLYHNT

DVVLAPYGEYWRQLRKICTLELLSVKKVKSFQSLREEECWNLVQEIKASGSGRPVNLSEN

VFKLIATILSRAAFGKGIKDQKELTEIVKEILRQTGGFDVADIFPSKKFLHHLSGKRARL

TSLRKKIDNLIDNLVAEHTVNTSSKTNETLLDVLLRLKDSAEFPLTSDNIKAIILDMFGA

GTDTSSSTIEWAISELIKCPKAMEKVQAELRKALNGKEKIHEEDIQELSYLNMVIKETLR

LHPPLPLVLPRECRQPVNLAGYNIPNKTKLIVNVFAINRDPEYWKDAEAFIPERFENSSA

TVMGAEYEYLPFGAGRRMCPGAALGLANVQLPLANILYHFNWKLPNGVSYDQIDMTESSG

ATMQRKTELLLVPSF

>sp|A0A2U1LIM9|NCPR1_ARTAN NADPH--cytochrome P450 reductase 1 OS=Artemisia annua OX=35608 GN=CPR1

PE=1 SV=2

MQSTTSVKLSPFDLMTALLNGKVSFDTSNTSDTNIPLAVFMENRELLMILTTSVAVLIGC

VVVLVWRRSSSAAKKAAESPVIVVPKKVTEDEVDDGRKKVTVFFGTQTGTAEGFAKALVE

EAKARYEKAVFKVIDLDDYAAEDDEYEEKLKKESLAFFFLATYGDGEPTDNAARFYKWFT

EGEEKGEWLEKLQYAVFGLGNRQYEHFNKIAKVVDEKLVEQGAKRLVPVGMGDDDQCIED

DFTAWKELVWPELDQLLRDEDDTSVATPYTAAVAEYRVVFHDKPETYDQDQLTNGHAVHD

AQHPCRSNVAVKKELHSPLSDRSCTHLEFDISNTGLSYETGDHVGVYVENLSEVVDEAEK

LIGLPPHTYFSVHTDNEDGTPLGGASLPPPFPPCTLRKALASYADVLSSPKKSALLALAA

HATDSTEADRLKFLASPAGKDEYAQWIVASHRSLLEVMEAFPSAKPPLGVFFASVAPRLQ

PRYYSISSSPKFAPNRIHVTCALVYEQTPSGRVHKGVCSTWMKNAVPMTESQDCSWAPIY

VRTSNFRLPSDPKVPVIMIGPGTGLAPFRGFLQERLAQKEAGTELGTAILFFGCRNRKVD

FIYEDELNNFVETGALSELVTAFSREGATKEYVQHKMTQKASDIWNLLSEGAYLYVCGDA

KGMAKDVHRTLHTIVQEQGSLDSSKAELYVKNLQMAGRYLRDVW

>sp|P0DTC9|NCAP_SARS2 Nucleoprotein OS=Severe acute respiratory syndrome coronavirus 2 OX=2697049

GN=N PE=1 SV=1

MSDNGPQNQRNAPRITFGGPSDSTGSNQNGERSGARSKQRRPQGLPNNTASWFTALTQHG

KEDLKFPRGQGVPINTNSSPDDQIGYYRRATRRIRGGDGKMKDLSPRWYFYYLGTGPEAG

LPYGANKDGIIWVATEGALNTPKDHIGTRNPANNAAIVLQLPQGTTLPKGFYAEGSRGGS

QASSRSSSRSRNSSRNSTPGSSRGTSPARMAGNGGDAALALLLLDRLNQLESKMSGKGQQ

QQGQTVTKKSAAEASKKPRQKRTATKAYNVTQAFGRRGPEQTQGNFGDQELIRQGTDYKH

WPQIAQFAPSASAFFGMSRIGMEVTPSGTWLTYTGAIKLDDKDPNFKDQVILLNKHIDAY

KTFPPTEPKKDKKKKADETQALPQRQKKQQTVTLLPAADLDDFSKQLQQSMSSADSTQA

>sp|P0DTC3|AP3A_SARS2 ORF3a protein OS=Severe acute respiratory syndrome coronavirus 2 OX=2697049

GN=3a PE=1 SV=1

MDLFMRIFTIGTVTLKQGEIKDATPSDFVRATATIPIQASLPFGWLIVGVALLAVFQSAS

KIITLKKRWQLALSKGVHFVCNLLLLFVTVYSHLLLVAAGLEAPFLYLYALVYFLQSINF

VRIIMRLWLCWKCRSKNPLLYDANYFLCWHTNCYDYCIPYNSVTSSIVITSGDGTTSPIS

EHDYQIGGYTEKWESGVKDCVVLHSYFTSDYYQLYSTQLSTDTGVEHVTFFIYNKIVDEP

EEHVQIHTIDGSSGVVNPVMEPIYDEPTTTTSVPL

>sp|P0DTC5|VME1_SARS2 Membrane protein OS=Severe acute respiratory syndrome coronavirus 2

OX=2697049 GN=M PE=3 SV=1

MADSNGTITVEELKKLLEQWNLVIGFLFLTWICLLQFAYANRNRFLYIIKLIFLWLLWPV

TLACFVLAAVYRINWITGGIAIAMACLVGLMWLSYFIASFRLFARTRSMWSFNPETNILL

NVPLHGTILTRPLLESELVIGAVILRGHLRIAGHHLGRCDIKDLPKEITVATSRTLSYYK

LGASQRVAGDSGFAAYSRYRIGNYKLNTDHSSSSDNIALLVQ

>sp|P0DTC7|NS7A_SARS2 ORF7a protein OS=Severe acute respiratory syndrome coronavirus 2 OX=2697049

GN=7a PE=1 SV=1

MKIILFLALITLATCELYHYQECVRGTTVLLKEPCSSGTYEGNSPFHPLADNKFALTCFS

TQFAFACPDGVKHVYQLRARSVSPKLFIRQEEVQELYSPIFLIVAAIVFITLCFTLKRKT

E
